# Mechanism of Stability and Transport of Chitosan-Stabilized Nano Zero-Valent Iron in Saturated Porous Media

**DOI:** 10.3390/ijerph18105115

**Published:** 2021-05-12

**Authors:** Dan Huang, Zhongyu Ren, Xiaoyu Li, Qi Jing

**Affiliations:** 1 Faculty of Architecture, Civil and Transportation Engineering, Beijing University of Technology, Beijing 100124, China; 19801355810@139.com (D.H.); renzhy@bjut.edu.cn (Z.R.); 2 Haihe River Water Conservancy Commission, Ministry of Water Resources, Tianjin 300171, China; 17810290991@139.com

**Keywords:** chitosan, nZVI, stability, transport, colloidal filtration theory, numerical simulation

## Abstract

Chitosan-stabilized nano zero-valent iron (CTS-nZVI) prepared by the liquid-phase reduction method has been shown to achieve a good dispersion effect. However, there has been little analysis on the mechanism affecting its stability and transport in saturated porous media. In this paper, settling experiments were conducted to study the stabilization of CTS-nZVI. The transport of CTS-nZVI in saturated porous media at different influencing factors was studied by sand column experiments. The stability mechanism of CTS-nZVI was analyzed from the point of view of colloidal stability by settling experiments and a zeta potential test. The theoretical model of colloidal filtration was applied for the calculation of transport coefficients on the basis of the column experiments data. Considering attachment–detachment effects, a particle transport model was built using HYDRUS-1D software to analyze the transport and spatial distribution of CTS-nZVI in a sand column.

## 1. Introduction

Nano zero-valent iron (nZVI) is a typical in situ remediation material for groundwater pollution [1,2]. As a reducing agent, nZVI can effectively remove pollutants such as chloroorganics, phenolic organics, organic dyes, pesticides, heavy metals and other pollutants [3,4,5,6]. However, nZVI particles can easily aggregate and form a deposit on porous media due to their poor dispersion in water, which reduces their mobility and activity [7,8]. Polymers, polyelectrolytes and surfactants can modify the surface of nZVI through physical deposition and chemical bonds [9,10,11,12], resulting in electrostatic and spatial repulsion, and overcoming the aggregation between particles caused by van der Waals forces and magnetic forces [13,14].

Chitosan, as the product of N-deacetylation of chitin, has the characteristics of biodegradability, biocompatibility and non-toxicity. Chitosan-supported nano zero-valent iron (CTS-nZVI) prepared by the liquid-phase reduction method has been shown to achieve a good dispersion effect [15,16]. Xu et al. [17] studied the degradation of trichloroethylene (TCE) by CTS-nZVI at different conditions. The results show that the use of chitosan can significantly improve the dispersion and stability of nZVI, and with the increase in the supporting ratio, the stability of nZVI increases gradually. When the pH is between 5 and 6, the supporting ratio is 26.4%, the mass ratio of nZVI to TCE is 40:1~50:1 and the removal rate of TCE by CTS-nZVI can reach 90%. Zhou et al. [18] successfully prepared chitosan-supported iron nickel bimetallic nanoparticles (CS-Fe-Ni) by using natural polymer chitosan as a supporting agent to enhance dispersion and stability. The settling spectrum of CS-Fe-Ni settling shows that the dispersion stability of CS-Fe-Ni nanoparticles was enhanced. The zeta potential test further confirmed that the increase in the negative charge of the particles enhanced the electrostatic repulsion force and improved the dispersion stability of CS-Fe-Ni. Column transport experiments showed that the transport ability of CS-Fe-Ni was improved. In recent years, there have been many research works on the modification technology of nZVI. By adding organic dispersants such as carboxymethyl starch cellulose (CMC), polyacrylic acid (PAA) and pectin into the nZVI suspension, the van der Waals force between particles decreases, the steric hindrance increases and the colloidal stability increases [19,20,21,22,23,24]. Previous studies have described the modification methods and mechanisms of CTS-nZVI thoroughly [16,25,26]. Our work explores the stability mechanism and transport behavior of CTS-nZVI in saturated porous media, from which the transport distance of CTS-nZVI is revealed, in order to provide an important theoretical and experimental basis for future in situ applications. The primary work of this paper includes: (1) preparation of CTS-nZVI by liquid-phase reduction and morphological and chemical/physical characterizations of CTS-nZVI; (2) stability of CTS-nZVI studied by settling experiments of CTS-nZVI with different influencing factors (pH, ionic strength and ion types) and zeta potential characterization; (3) exploration of the transport properties of CTS-nZVI in saturated porous media at different factors (pH, ionic strength, ion types and injection velocities) through sand column experiments; (4) further analysis of the transport mechanism of CTS-nZVI through the colloidal filtration theory and simulation of solute transport.

## 2. Materials and Methods

### 2.1. Materials

All the chemical reagents used in this study were of analytical grade. Chitosan (deacetylation degree ≥ 95%) was purchased from Shanghai Aladdin Biotechnology Co., Ltd. (Shanghai, China); FeSO_4_·7H_2_O, NaBH_4_, NaCl, CaCl_2_ and KCl were purchased from Tianjin Fuchen Chemical Reagent Factory (Tianjin, China); glacial acetic acid, NaOH, HCl and HNO_3_ were purchased from Beijing Chemical Plant (Beijing, China). High-purity nitrogen (industrial grade) was purchased from Beijing Shunchi East Ring Dry Ice Business Center (Beijing, China). All solutions in this study were prepared with deionized water (DI).

Quartz sand with a particle size of 0.5~1 mm was selected as porous media. The quartz sand was pickled in hydrochloric acid, then cleaned with DI water for many times until the pH of the supernatant was close to DI water and then it was ready for use after air drying.

### 2.2. Characterization and Analytical Methods

The surface morphology and structure of CTS-nZVI were observed using a scanning electron microscope (FE-SEM, JEOL JSM-7001M, Tokyo, Japan). The binding energy of CTS-nZVI was recorded by X-ray photoelectron spectroscopy (Thermo Fisher ESCALAB 250Xi, Waltham, MA, USA). Functional groups of CTS-nZVI were analyzed by a Fourier transform infrared spectrometer (Shimadzu IRAffinity-1S, Tokyo, Japan). The settling rate of CTS-nZVI in solution and the concentration of iron were quantified by UV–Vis spectroscopy (UNICO 2082S UV/VIS, Shanghai, China). The average size and size distribution of CTS-nZVI were measured by the dynamic light scattering technique (Brookhaven Instruments Corporation 90 PLUS, New York, NY, USA). Zeta potential of CTS-nZVI was determined using a Brookhaven 90 plus zeta potential analyzer (Brookhaven Instruments Corporation 90 PLUS, New York, NY, USA). The pH was measured using a pH-meter (Mettler Toledo MP 220, Zurich, Switzerland).

In this study, the settling curves of different pH and ionic strengths were fitted by Origin 2018 software. The standard deviation calculation of the parallel test and the diagram in this paper were plotted using Origin 2018 software. The numerical model of water flow and solute transport in porous media was established by using software version 4.0 of HYDRUS-1D.

### 2.3. Preperation of CTS-nZVI

In this study, CTS-nZVI was prepared by the liquid-phase reduction method. An amount of 1 g chitosan was added into 200 mL of 0.05 mol/L HNO_3_ solution, placed in a shaker (Thermostatic oscillator (DSHZ-300A), Jiangsu, China) at a constant temperature of 40 °C and vortexed for 4 h at 200 r/min to obtain 0.5% chitosan solution. An amount of 2.978 g FeSO_4_·7H_2_O was dissolved in 100 mL DI water, followed by adding 30 mL of the above 0.5% chitosan solution through a 0.22 μm microporous membrane, and then oxygen was removed with N_2_ for 30 min and stirred for 10 min to make it fully mixed. At the same time, 100 mL 0.535 mol/L NaBH_4_ solution was added into the mixed solution, and black iron nanoparticles were formed after 90 min of reaction. The reaction equation is as follows [18]:

(1) $${\mathrm{FeSO}}_{4}+2{\mathrm{NaBH}}_{4}+6H_{2}O\;\overset{Chitosan}{\to }\;{\mathrm{Fe}}^{0}+2B(\mathrm{OH})3_{\;}+{\mathrm{Na}}_{2}{\mathrm{SO}}_{4}+7H_{2}↑$$

The prepared CTS-nZVI was filtered by a vacuum suction filter and cleaned with deoxygenated water for three times to remove the impurities.

### 2.4. Settling Experiment

The CTS-nZVI suspension with a concentration of 150 mg/L was prepared, and the pH range was adjusted to 6~8 using 1% NaOH and 1% HCl solution (the pH of groundwater is generally within this range [27]). After 10 min of ultrasonic treatment (Kunming Ultrasonic Instrument Co., Ltd. KQ-300DE, Kunming, China), the samples were placed in a colorimetric dish, the absorbance was determined by a UV spectrophotometer at 508 nm and the settling curves of CTS-nZVI were drawn.

Water solution of CTS-nZVI with 150 mg/L concentration was prepared first. Then, an appropriate amount of NaCl (CaCl_2_) was added to make the corresponding concentration of NaCl (CaCl_2_) reach 0 mM, 30 mM and 100 mM (0 mM, 10 mM and 33 mM, respectively), and the ionic strength was 0 mM, 30 mM and 100 mM. After treatment with an ultrasonic dissolver for 10 min, the sample was placed in the cuvette, and its absorbance was determined by a spectrophotometer at 508 nm [28]. The settling curves of CTS-nZVI were drawn. A zeta potential analyzer was used to measure the zeta potential of CTS-nZVI under different influencing factors. Zeta potential was determined by a parallel test and repeated three times.

### 2.5. Sand Column Experiment

The transport experimental device is shown in Figure S1. It is mainly composed of three parts: CTS-nZVI suspension mixer, peristaltic pump and quartz sand column.

The total length of the plexiglass column is 18 cm. In the column, 1.5-cm-high glass beads (3 mm in diameter) are filled at the inlet and outlet of both ends to ensure uniform water distribution. Quartz sand with a particle size of 1–2 mm after pickling was filled in the middle. During the filling process, quartz sand was added slowly, and the glass column was continuously knocked. A peristaltic pump was used to inject 10 pore volumes (PVs) deoxygenated water to establish chemical and hydrochemical equilibration between the sand and water, eventually removing residual colloids. On the other hand, it also plays a role in flushing and removing impurities from the quartz sand [29].

In order to investigate the effects of injection speed, pH, ionic strength and ion species on the transport of CTS-nZVI, the following tests were carried out.

The concentration of the CTS-nZVI suspension was 150 mg/L, and 3 PVs suspension was pumped by the peristaltic pump at the injection rates of 9 mL/min and 18 mL/min, and then 5 PVs deoxidized water was injected at the corresponding injection rate. Samples were taken at the outlet of the sand column, and then breakthrough and flushing curves were drawn. In order to reduce the aggregation and precipitation of CTS-nZVI, the suspension was treated by ultrasound about 10 min before and during injection. After the column experiment was completed, the quartz sand in the test column was taken out and soaked in a certain volume of hydrochloric acid to test the concentration of iron in the solution (spectrophotometry) [28], in order to convert the solid concentration at different spatial positions in the media.

## 3. Results

### 3.1. Characterization of CTS-nZVI

**Figure 1:**
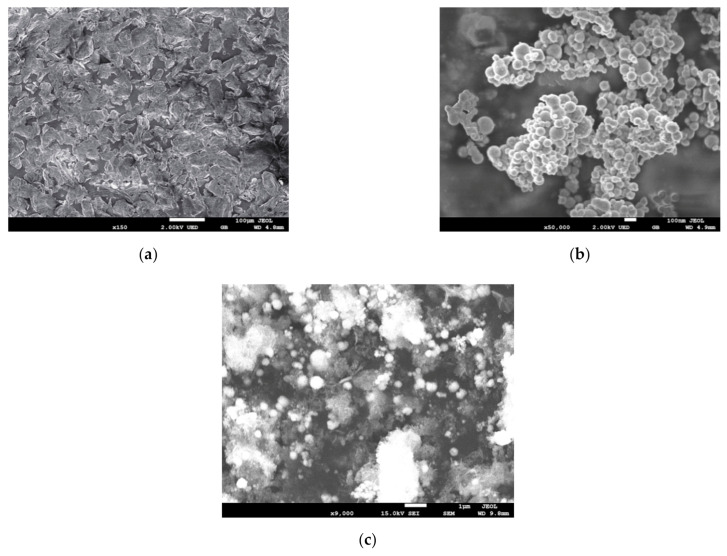
SEM images of the surface of (**a**) bare chitosan, (**b**) bare nZVI and (**c**) CTS-nZVI prepared by liquid-phase reduction.

The surface morphologies of chitosan (Figure 1a), bare nZVI (Figure 1b) and CTS-nZVI (Figure 1c) were observed by SEM. The results show that the particle size of the bare nZVI is about 100 nm, and the particle aggregation is obvious. From the SEM images of CTS-nZVI, it can be observed that the average particle size of CTS-nZVI is significantly larger than that of bare nZVI. This also indicates that the chitosan attached to the surface of nZVI increases the steric hindrance and reduces the aggregation of nanoparticles.

**Figure 2:**
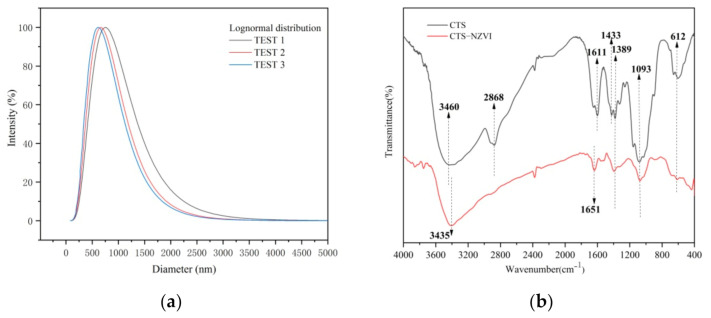
(**a**) Particle size distribution of CTS-nZVI, (**b**) FTIR spectra of the bare chitosan and CTS-nZVI.

As shown in Table 1, the particle size of CTS-nZVI was measured by a DLS laser particle size analyzer. The total measurements of the sample were set to three. The results show that the average effective particle size is 665 nm, and the average polydispersity index is 0.281. The reliability of the experimental results was determined by summarizing the statistical report date. It was confirmed that the measurement results are valid and reliable when the average count rate is faster than 50 kcps, the baseline index is higher than 5 and data retention exceeds 95%. The particle size distribution of CTS-nZVI is determined in Figure 2a.

Fourier transform infrared spectroscopy was used to analyze chitosan and CTS-nZVI. As shown in Figure 2b, in the infrared spectrum of chitosan, there is a wide peak formed by the overlapping stretching vibration absorption peak of O-H and the stretching vibration absorption peak of N-H around 3460 cm^−1^, a C-H stretching vibration absorption peak of the methyl or methylene group near 2868 cm^−1^, an N-H straining vibration absorption peak of the amino group (-NH_2_) near 1611 cm^−1^ and a straining vibration absorption peak of C-CH_3_ around 1433 cm^−1^ and 1389 cm^−1^. There is also a stretching vibration absorption peak of C-OH near 1093 cm^−1^ [30].

**Figure 3:**
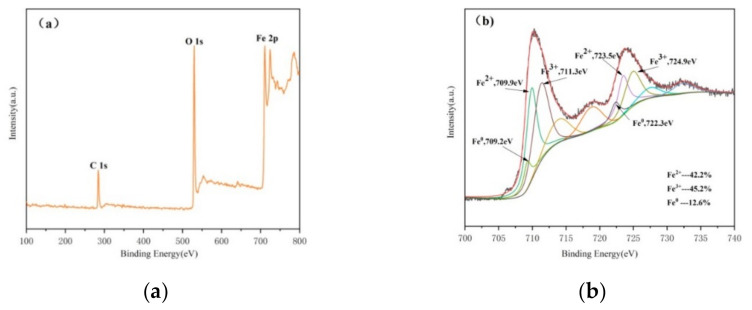
The XPS spectra of CTS-nZVI: (**a**) survey spectra, (**b**) Fe2p spectra.

As shown in Figure 3a, binding energies near 284.08 eV and 530.08 eV are found, which are attributed to C1s and O1s, respectively, and the electron binding energy fe2p at 711.08 eV and 724.08 eV can be observed, which is the photoelectron energy measured when 2p orbital electrons of the iron atom are excited. In order to better explore the existing form of iron in CTS-nZVI, XPS was used to scan the photoelectron peak of iron (Figure 3b), where there are 2p3/2 and 2p1/2 peaks corresponding to Fe^2+^ at the binding energies of 709.9 eV and 723.5 eV, and 2p3/2 and 2p1/2 corresponding to Fe^3+^ at 711.3 eV and 724.9 eV. The characteristic peaks corresponding to Fe^0^ are found at the binding energies of 709.2 eV and 722.3 eV, indicating that Fe^0^ was partially oxidized to Fe^2+^ and Fe^3+^ during the preparation and characterization.

### 3.2. Effect of pH on Settling and Transport

#### 3.2.1. Effect of pH on Settling

**Figure 4:**
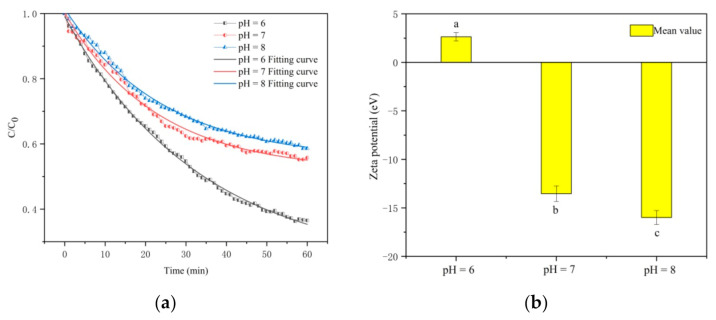
(**a**) The settling curves and (**b**) zeta potential mean values of CTS-nZVI at different pH (the letters a, b and c show the statistically significant differences between variables).

The settling curves of CTS-nZVI at different pH were obtained by measuring the absorbance with a spectrophotometer, as shown in Figure 4a. Table 2 shows the better fitting results of the settling curves fitted with an exponential function by Origin software at different pH. The asymptote occurs when the two actions of settling and diffusion are in balance. The relative concentrations of CTS-nZVI corresponding to the equilibrium of the fitting curves at different pH of 6, 7 and 8 are 0.2251, 0.5163 and 0.5514, respectively, i.e., with the enhancement in pH, the relative equilibrium concentration increases, indicating the better stability of CTS-nZVI. The settling rates of CTS-nZVI with an interval of 10 min at different pH are calculated in Table 3, showing the settling rates decrease with the increase in pH and the time.

The settling equilibrium time of CTS-nZVI is defined as the settling rate less than 0.0001 min^−1^. Through the calculation of the fitting curve, the settling equilibrium time for CTS-nZVI at different pH is determined to be 160–170 min, 110–120 min and 110–120 min. In conclusion, the stability of CTS-nZVI at pH 7 and pH 8 was better than that at pH 6.

As shown in Figure 4b, pH has significant effects on the zeta potential of CTS-nZVI (the error bars represent the standard deviation of three measurements, and the data, SD and labels of each group are shown in Table S1). With the increase in pH from 6 to 8, the zeta potential absolute values of CTS-nZVI gradually increase. The zeta potential changes from 2.4 at pH 6 to −13.53 mV at pH 7, decreasing by 5.13 times. That the zeta potential value at pH 6 is positive may be due to protonation of amino groups in chitosan molecules under acidic conditions, which makes CTS-nZVI particles positively charged [16].

#### 3.2.2. Effect of pH on Transport

**Figure 5:**
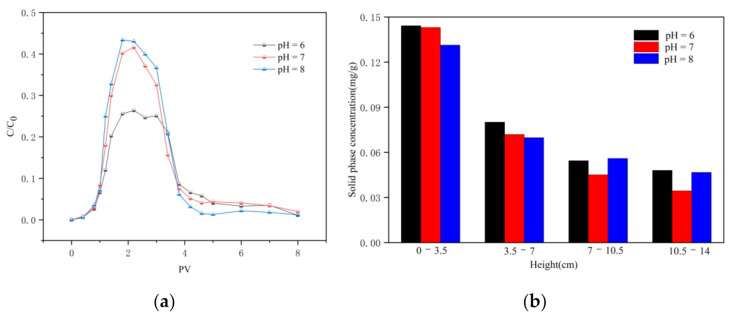
(**a**) The breakthrough and flushing curves of CTS-nZVI at different pH, and (**b**) the solid content distribution of CTS-nZVI at different pH in porous media.

Breakthrough and flushing curves of CTS-nZVI nanoparticles at different pH are provided in Figure 5a. The results show that with the increase in pH (6–8), the relative effluent concentration increased, which is consistent with the effect of pH on the stability of CTS-nZVI. With the increase in pH, the stability of CTS-nZVI nanoparticles gradually increases, which reduces the deposition of CTS-nZVI on the surface of porous media, in order to enhance the transport of CTS-nZVI.

Figure 5b shows the solid concentration distribution of CTS-nZVI in porous media after column experiments. The results are shown in Table 4.

It can be seen from Figure 5b and Table 4 that the solid phase content of CTS-nZVI is the highest at the injection end. With the increase in distance, the solid phase content decreases gradually and reaches its lowest value at the outlet. At pH 6, the retention of CTS-nZVI is the highest, and at pH 8, the spatial distribution of CTS-nZVI in the sand column is relatively uniform.

### 3.3. Effect of Ionic Strength and Ion Species on Settling and Transport

#### 3.3.1. Effect of Ionic Strength and Ion Species on Settlement

**Figure 6:**
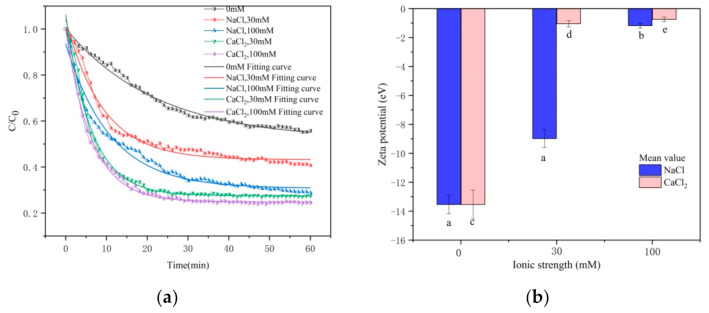
(**a**) The settling curves and (**b**) zeta potential of CTS-nZVI at different ionic strengths and ionic species (the letters a, b, c, d and e show the statistically significant differences between variables).

The settling curves of CTS-nZVI at different ionic strengths and ionic species are shown in Figure 6a, and the curves fitted with an exponential function by Origin software are shown in Table 5.

According to Table 5, the settling curves satisfy the law of the exponential function. It can be seen from the asymptote of the fitting equation that the equilibrium concentration of CTS-nZVI decreases with the increase in ionic strength. At the same ionic strength, the settling equilibrium concentration of Ca^2+^ is lower than that of Na^+^. The settling rates of CTS-nZVI under different conditions are calculated as shown in Table 6. The greater the ionic strength, the faster the settling rate. The settling rate of Ca^2+^ is higher than that of Na^+^ at the same ionic strength. As the ionic strength increases from 0 to 30 mM, the settling is in equilibrium at the time of 50–60 min. In conclusion, the lower the ionic strength, the better the stability of CTS-nZVI. At the same ionic strength, Ca^2+^ has a more negative influence on the stability of CTS-nZVI than Na^+^. 

Ionic strength and ion species effect the zeta potential of CTS-nZVI, which are provided in Figure 6b (the data, SD and labels of each group are shown in Table S2). With the increase in ionic strength, the zeta potential absolute value of CTS-nZVI gradually decreases. By comparing the zeta potential of different types of ions, Ca^2+^ can significantly reduce the zeta potential absolute value. The results show that Ca^2+^ has more influence on the zeta potential of CTS-nZVI than Na^+^.

#### 3.3.2. Effect of Ionic Strength and Ion Species on Transport

**Figure 7:**
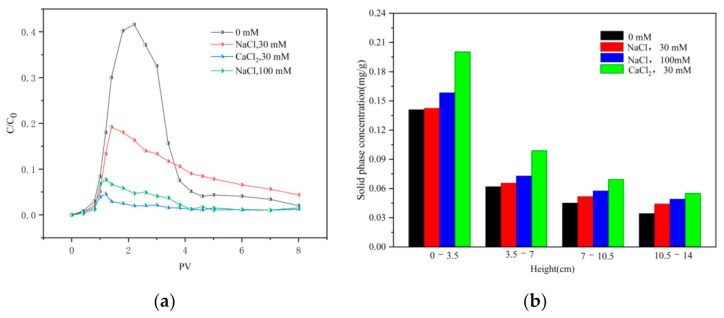
(**a**) The breakthrough and flushing curves of CTS-nZVI at different ionic strengths and ionic species, and (**b**) the solid concentration distribution of CTS-nZVI at different ionic strengths and ionic species in porous media.

Breakthrough and flushing curves of CTS-nZVI with different ionic strengths and ionic species are given in Figure 7a. The results show that the relative effluent concentration decreases with the increase in ionic strength. By comparing the concentration profiles of different types of ions, Ca^2+^ has a stronger effect on reducing the mobility of CTS-nZVI than Na^+^.

This is consistent with the influence of the ion strength and ion species on the settling experiment, that is, with the increase in ionic strength, the stability of CTS-nZVI gradually decreases, which enhances its deposition on the surface of porous media and reduces its mobility. Compared with Na^+^, Ca^2+^ significantly reduces the stability of CTS-nZVI and increases the deposition of CTS-nZVI on the surface of porous media, leading to an increase in CTS-nZVI mobility. Figure 7b shows the solid content distribution of CTS-nZVI in porous media after column experiments. The results are shown in Table 7.

It can be seen from Figure 7b and Table 7 that the solid phase content of CTS-nZVI is the highest at the column inlet, and it gradually decreases with the increase in transport distance. The increase in ionic strength improves the retention of CTS-nZVI in the sand column and results in deposition at the surface, i.e., decreasing the transport of CTS-nZVI in the saturated porous media. At the same ionic strength, the addition of Ca^2+^ reduces the stability of CST- nZVI more than Na^+^.

### 3.4. Effect of Injection Velocity on Transport

**Figure 8:**
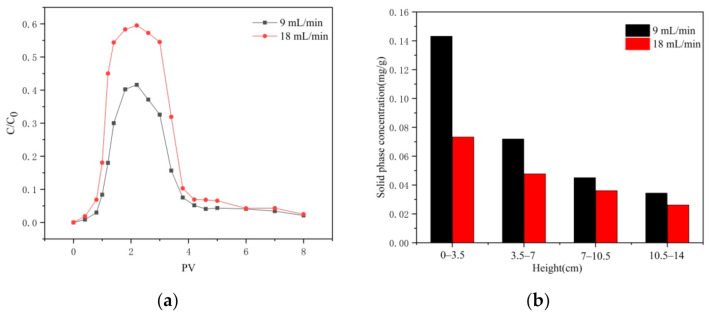
(**a**) The breakthrough and flushing curves of CTS-nZVI at different injection velocities, and (**b**) the solid concentration distribution of CTS-nZVI at different injection velocities in porous media.

The breakthrough and flushing curves of CTS-nZVI at different injection velocities are illustrated in Figure 8a. It can be seen from the curve that the relative outflow concentration increases with the increase in injection velocity. This shows that a larger hydrodynamic shear force can effectively reduce the deposition of CTS-nZVI in porous media, which is conducive to enhancing particle transport [31,32].

Figure 8b shows the distribution of the solid phase concentration of CTS-nZVI in different layers of porous media at different injection rates after column experiments. From the column inlet to the outlet, they are named layer 1 to layer 4. The results of the residual iron content and total residual iron rate of each layer are shown in Table 8.

According to Figure 8b and Table 8, it can be seen that the distribution of CTS-nZVI in porous media is ununiform. The content of the solid phase is the highest at the column inlet and gradually decreases with the increase in distance. The amount of retained iron at the injection rate of 18 mL/min is significantly less than that of 9 mL/min, indicating that the transport of CTS-nZVI was enhanced and the retained iron in the sand column was reduced by increasing the injection rate.

## 4. Discussion

### 4.1. Mechanism Analysis of Influence on Stability

In this study, the stability of CTS-nZVI was analyzed from the perspective of colloidal stability. Brownian motion, the charge of dispersed particles and solvation are three important reasons for the stability of colloidal particles [33].

The charge of colloidal particles is the main factor of colloidal stability. The solid and liquid phases in contact often have opposite sign charges due to ion deposition or dissociation [33]. When the concentration of the electrolyte increases, the concentration of opposite ions in the media increases, meaning that the surface charge of the solid is neutralized, and the zeta potential decreases in numerical value [33]. Therefore, with the increase in ionic strength, the absolute value of the zeta potential of CTS-nZVI decreases gradually, which leads to the decline in stability. Similarly, the elevation in pH increases the absolute value of the zeta potential of CTS-nZVI gradually, which enhances the amount of surface charge, resulting in an increase of repulsion forces between particles [34], thus gradually improving the stability of CTS-nZVI.

Compared with Na^+^, Ca^2+^ has more influence on the stability of CTS-nZVI. This can be explained by the Schulze–Hardy rule [31]. The Schulze–Hardy rule states that the ions in the electrolyte that can cause the colloidal particles to aggregate are the ions opposite the charged sign of colloidal particles, the counter ions. The higher the valence number of the counter ions, the greater the agglomeration capacity. The higher the valence number of Ca^2+^ than Na^+^, the easier it is for CTS-nZVI to agglomerate. Therefore, compared with Na^+^, Ca^2+^ reduces the stability of CTS-nZVI.

### 4.2. Colloidal Filtration Theory

The theoretical model of colloidal filtration depends on two parameters: (1) $\eta_{0}$: single particle capture coefficient, and (2) α: adhesion coefficient. $\eta_{0}$ can be calculated by an empirical formula, which is mainly affected by three factors [35]: particle interception, gravity settlement and Brownian diffusion; the adhesion coefficient is the adhesion efficiency between nanoparticles and media, which depends on the interaction between particles and media.

The contact efficiency of a single collector is calculated as follows [36]:

(2) $$\eta_{0}=\eta_{I}+\eta_{G}+\eta_{D}$$

(3) $$\eta_{I}=0.55A_{S}N_{R}^{1.55}N_{Pe}^{-0.125}N_{vdW}^{0.125}$$

(4) $$\eta_{G}=0.475N_{R}^{-1.35}N_{Pe}^{-1.11}N_{vdW}^{0.053}N_{gr}^{1.11}$$

(5) $$\eta_{D}=2.4A_{S}^{1/3}N_{R}^{-0.081}N_{Pe}^{-0.715}N_{vdW}^{0.052}$$

The meanings of the parameters are shown in Table S3.

Colloidal filtration theory can be used to predict the maximum transport distance of colloidal particles [37]:

(6) $$L_{max}=-\frac{2d_{c}}{3\left(1-\theta \right)\alpha \eta_{0}}\ln\left(0.001\right)$$

Table S4 shows the contact efficiency of a single collector at different $\eta_{0}$, *α* and $L_{max}$.

According to Table S4, the weight ratio of gravity settlement, Brownian diffusion and interception is 118:29:1~254:48:1. Therefore, under the experimental conditions of this study, the gravity settlement is the main factor of CTS-nZVI deposition.

With the increase in pH, the adhesion coefficient α decreases and the maximum transport distance increases, which indicates that the increase in pH is beneficial to the transport of CTS-nZVI in saturated porous media. With the increase in ionic strength, the adhesion coefficient α increases and the maximum transport distance decreases significantly, which indicates that the increase in ionic strength is not conducive to the transport of CTS-nZVI in saturated porous media, and the ionic strength has a great influence on the transport of iron nanoparticles. Compared with Na^+^, Ca^2+^ has a stronger inhibitory effect on the transport of iron nanoparticles. With the increase in the water flow velocity, the maximum transport distance of iron nanoparticles increases significantly.

### 4.3. Simulation of Solute Transport

In this study, a numerical model of water flow and solute transport in porous media was established by using HYDRUS-1D software. The simulation includes two processes. One is the transport process of the nZVI suspension after being pumped into the saturated sand column from bottom to top, and the other is the transport process of nZVI in the saturated sand column under subsequent flushing by deoxygenated water. The unit of time is min, the unit of mass is μg and the unit of length is cm.

The one-dimensional equilibrium flow is described by the Richards equation [38,39]:

(7) $$\frac{\partial \theta }{\partial t}=\frac{\partial }{\partial x}\left[K\;\left(\frac{\partial h}{\partial x}+cos\alpha \right)\right]\;-\;S$$

where *H* is the water head, cm; *θ* is the soil volume moisture content; *t* is the time, min; *s* is the source and sink term, min^−1^; *α* is the angle between the flow direction and the vertical direction (*α* = 0° represents vertical flow, 90° represents horizontal flow, 0° < *α* < 90° represents inclined flow). In this study, *α* = 0°; *X* is the space position, cm; and *K* is the unsaturated hydraulic conductivity, cm min^−1^.

The initial condition of the flow model is the whole saturation state, that is, the initial water content is the saturated water content. The upper and lower boundaries of the flow model are constant flow boundaries.

In this study, a solute transport equation based on the attachment–detachment model was established to simulate the transport of CTS-nZVI in saturated porous media. The expression of the equation is as follows [40]:

(8) $$\frac{\partial \left(\theta C\right)}{\partial t}=\;\frac{\partial }{\partial x}(\theta D\frac{\partial C}{\partial x})\;-\;\frac{\partial }{\partial x}(vC)-\rho \frac{\partial S_{1}}{\partial t}-\rho \frac{\partial S_{2}}{\partial t}$$

where *θ* is the soil volume moisture content; *C* is the solute liquid phase concentration, mg·mL^−1^; *S* is the solute content in solid media, ug·g^−1^; the subscripts 1 and 2 represent the two kinetic adsorption sites; *D* is the dispersion coefficient, cm^2^·min^−1^; *V* is the Darcy velocity, cm·min^−1^; *X* is the spatial position, cm; ρ is the dry bulk density of porous media, g·cm^−3^; *t* is the time, min. In this equation, two source and sink terms $\;\rho \frac{\partial S_{1}}{\partial t}\;\mathrm{and}\;\rho \frac{\partial S_{2}}{\partial t}$ are used to represent the interaction of nZVI with the solid matrix.

The initial condition of the model is that the solute concentration at each position of the sand column is 0 at the initial time. The lower boundary of the model is defined as concentration flux, and the given concentration boundary and the upper boundary are concentration flux boundaries.

This simulation imitates the transport of nZVI nanoparticles in saturated porous media at two different injection rates. The model parameters are shown in Table S5.

**Figure 9:**
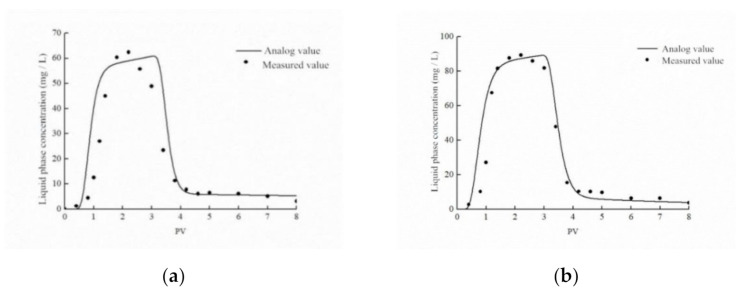

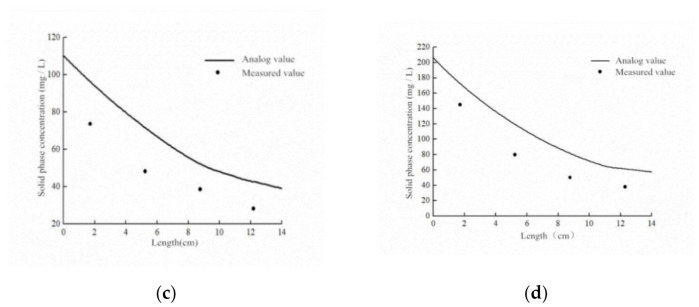
Comparison diagrams of measured outflow concentration and simulated values of attachment–detachment model at different injection velocities: (**a**) 9 mL/min, (**b**) 18 mL/min. Comparison of measured values of solid phase concentration at four different depths and simulated values of attachment–detachment model at different injection velocities: (**c**) 9 mL/min, (**d**) 18 mL/min.

From the observation of Figure 9a,b, it can be seen that the simulation value of the attachment–detachment model in the simulation of the CTS-nZVI injection and subsequent flushing process is consistent with the measured value at the outlet of the sand column, which can better describe the transport of CTS-nZVI in the sand column.

It can be seen from Figure 9c,d, that the trend of the simulation value of the solid phase concentration at different depths is consistent with the measured value, indicating that the model is generally and vaguely fitted to simulate the spatial distribution of the solid phase concentration of CTS-nZVI at different depths. It is also found that there is a certain difference between the simulated value and the measured value, which may be due to the fact that the model simulation is an ideal situation, and there is a certain error in measuring the solid concentration at different depths in the sand column. Generally speaking, the attachment–detachment model can well simulate the transport and spatial distribution of CTS-nZVI in the sand column.

A Nash–Sutcliffe simulation efficiency coefficient (NSC) evaluation model was used to evaluate the results, and the calculation formula is as follows [41]:

(9) $$NSC=1\;-\;\frac{\sum {\left(X_{obs}\;-\;X_{model}\right)}^{2}}{\sum {\left(X_{obs}\;-\;{\bar{X}}_{obs}\right)}^{2}}$$

where $X_{obs}$ is the observed or measured value, $X_{model}$ is the calculated value of the model, and ${\bar{X}}_{obs}$ is the arithmetic mean of the measured values. When the calculated value of the model is equal to the actual monitoring value, the simulation effect is the best. Generally, the NSC is between 0 and 1, and the larger the NSC, the better the matching degree between the calculated value and the observed value. The NSC was 0.70 at 9 mL/min and 0.86 at 18 mL/min. In general, the attachment–detachment model can well simulate the transport of CTS-nZVI in the sand column.

**Figure 10:**
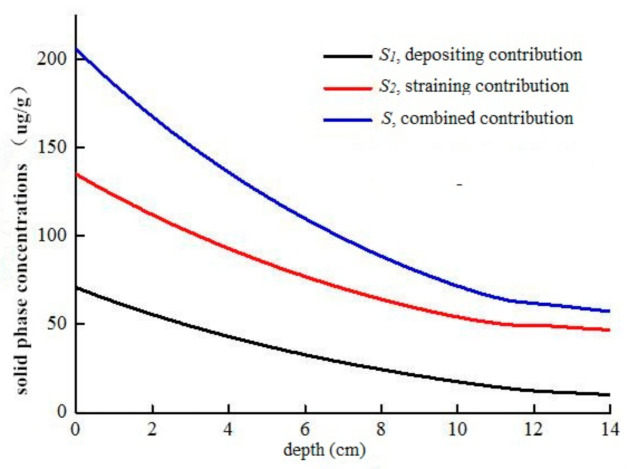
The contribution curves of deposition, straining and their combined effects on solid distribution with injection velocity of 9 mL/min.

In the attachment–detachment model, two source and sink terms $\rho \frac{\partial S_{1}}{\partial t}\;\mathrm{and}\;\rho \frac{\partial S_{2}}{\partial t}$ are used to represent the reaction of CTS-nZVI with the solid matrix. $S_{1}\;\mathrm{and}\;S_{2}$ are the deposition and straining (this means that when some pores in the aquifer are smaller than some critical sizes, the particles cannot pass through and stay in the aquifer) of particles. The attachment–detachment model describes the attachment of CTS-nZVI in the sand column through the combination of the above two actions. At the same time, it is considered that the CTS-nZVI attached to the porous media will be detached with the flow of water, and the detachment rate is determined by the parameter $k_{det}$. Figure 10 shows the contribution curves of deposition, straining and their combined effects on the solid distribution with an injection velocity of 9 mL/min. It is determined by the total solid concentration (S) in the porous media, the solid concentration of CTS-nZVI nanoparticles deposited to the porous media due to the effect of particle attachment and detachment (*S*_1_) and the solid concentration of CTS-nZVI nano iron retained in the porous media due to physical straining (*S*_2_). It can be seen from Figure 10 that the amount of CTS-nZVI retained in the porous media due to straining is greater than that attached to the porous media, accounting for 70.52% and 29.48% of the total iron content in the column, respectively. Straining is an important factor affecting the transport of CTS-nZVI.

## 5. Conclusions

This study systemically explored the mechanisms of stability and transport of CTS-nZVI in saturated porous media through laboratory experiments, theoretical calculation and simulation modeling. The following conclusions are drawn:

(1) The characterization results exhibit that chitosan improves antioxidation and inhibits the aggregation of nZVI particles.
(2) With the higher pH and the lower ionic strength, the stability of nZVI is better. At the same ionic strength, Ca^2+^ has a more negative influence on the stability of CTS-nZVI than Na^+^. The charge amount of CTS-nZVI changes the repulsive potential energy between particles, which changes the stability of CTS-nZVI.
(3) A series of sand column experiments show that the transport of CTS-nZVI in saturated porous media is related to its stability. When the pH is higher and the ionic strength is lower, the mobility of nZVI is better. At the same ionic strength, Ca^2+^ has a more negative influence on the mobility of CTS-nZVI than Na^+^. As the larger fluid shear force can promote the mobility of nanoparticles, CTS-nZVI exhibits an enhanced transport in saturated porous media with the increase in injection velocity.
(4) According to the colloidal filtration theory, gravity settlement is an important factor affecting the deposition of CTS-nZVI. Transport behaviors of CTS-nZVI are described by the nanoparticles’ spatial distribution in sand columns using an attachment–detachment model in HYDRUS-1D. The simulation results show that straining is an important factor affecting the transport of CTS-nZVI.

Our work reveals the stability mechanism and the transport distance of CTS-nZVI, which will provide an important theoretical and experimental basis for the practical application of in situ injection technology using CTS-nZVI during groundwater remediation.

## Figures and Tables

**Table 1:** Summary statistical report of particle size measurement.

NO.	Eff. Diam. (nm)	Polydispersity	Baseline Index	Count Rate (kcps)	Data Retained (%)	Diffusion Coeff. (cm^2^/s)
TEST 1	723.00	0.299	8.6	421.5	97.07	6.535 × 10^−9^
TEST 2	662.00	0.276	9.4	420.5	99.34	7.380 × 10^−9^
TEST 3	610.00	0.267	6.9	513.1	98.29	2.947 × 10^−9^
Mean	665.00	0.281	8.3	451.7	98.23	5.620 × 10^−9^

**Table 2:** The mathematical description of the CTS-nZVI settling curves at different pH.

Influence Factor	Expression (y is Relative Concentration, x is Time)	R^2^	Sum of Squares of Residuals	Asymptote
pH = 6	y = 0.2251 + 0.7655 × exp(−0.0298x)	0.9986	0.00005	y = 0.2251
pH = 7	y = 0.5163 + 0.4858 × exp(−0.0443x)	0.9893	0.00018	y = 0.5163
pH = 8	y = 0.5514 + 0.4635 × exp(−0.0415x)	0.9949	0.00008	y = 0.5514

**Table 3:** Calculations of settling rate from fitting curves.

pH	Time (min)	Δ(C/C_0_)	Settling Rate (min^−1^)
6	0~10	0.19727	0.01973
	10~20	0.14643	0.01464
	20~30	0.10870	0.01087
	30~40	0.08069	0.00807
	40~50	0.05989	0.00600
	50~60	0.04446	0.00445
7	0~10	0.17386	0.01738
	10~20	0.11164	0.01116
	20~30	0.07169	0.00717
	30~40	0.04603	0.00460
	40~50	0.02956	0.00296
	50~60	0.01898	0.00190
8	0~10	0.15743	0.01574
	10~20	0.10396	0.01040
	20~30	0.06865	0.00686
	30~40	0.04533	0.00453
	40~50	0.02993	0.00299
	50~60	0.01977	0.00198

**Table 4:** Retain ratio and retained iron content at different pH.

pH	Injection Iron (mg)	Retained Iron (mg)	Retain Ratio
6	18.25	12.12	66.41%
7	18.25	10.93	59.89%
8	18.25	11.25	61.64%

**Table 5:** The mathematical descriptions of the CTS-nZVI settling curves at different ionic strengths and ionic species.

Influencing Factor	Expression (y is Relative Concentration, x is Time)	R^2^	Sum of Squares of Residuals	Asymptote
0 mM	y = 0.5163 + 0.4858 × exp(−0.0443x)	0.9893	0.00018	y = 0.5163
NaCl, 30 mM	y = 0.4318 + 0.6092 × exp(−0.1101x)	0.9828	0.00040	y = 0.4318
NaCl, 100 mM	y = 0.3068 + 0.6280 × exp(−0.0916x)	0.9802	0.00053	y = 0.3068
CaCl_2_, 30 mM	y = 0.2748 + 0.7863 × exp(−0.1603x)	0.9920	0.00025	y = 0.2748
CaCl_2_, 100 mM	y = 0.2482 + 0.7504 × exp(−0.1590x)	0.9966	0.00010	y = 0.2483

**Table 6:** Calculations of settling rate under different ionic strengths and ionic species.

Influencing Factor	Time (min)	Δ(C/C_0_)	Settling Rate (min^−1^)	Influencing Factor	Time (min)	Δ(C/C_0_)	Settling Rate (min^−1^)
0 mM	0~10	0.17386	0.01739				
	10~20	0.11164	0.01116				
	20~30	0.07169	0.00717				
	30~40	0.04603	0.00460				
	40~50	0.02956	0.00296				
	50~60	0.01898	0.00190				
NaCl, 30 mM	0~10	0.40662	0.04066	NaCl, 100 mM	0~10	0.37673	0.03767
	10~20	0.13522	0.01352		10~20	0.15074	0.01507
	20~30	0.04496	0.04496		20~30	0.06031	0.00603
	30~40	0.01495	0.00149		30~40	0.02413	0.00241
	40~50	0.00497	0.00050		40~50	0.00966	0.00097
	50~60	0.00165	0.00017		50~60	0.00386	0.00039
CaCl_2_, 30 mM	0~10	0.62802	0.62802	CaCl_2_, 100 mM	0~10	0.59737	0.05974
	10~20	0.12642	0.12642		10~20	0.12182	0.01218
	20~30	0.02545	0.02545		20~30	0.02484	0.00248
	30~40	0.00512	0.00512		30~40	0.00507	0.00051
	40~50	0.00103	0.00103		40~50	0.00103	0.00010
	50~60	0.00021	0.00021		50~60	0.00021	0.00002

**Table 7:** Retain ratio and retained iron content at different injection rates.

Influence Factor	Injection Iron (mg)	Retained Iron (mg)	Retain Ratio
0 mM	18.25	10.93	59.89%
NaCl, 30 mM	18.25	12.54	68.71%
NaCl, 100 mM	18.25	15.28	83.72%
CaCl_2_, 30 mM	18.25	15.73	86.19%

**Table 8:** Retain ratio and retained iron content at different injection velocities.

Injection Velocity (mL/min)	Injection Iron (mg)	Retained Iron (mg)	Retain Ratio
9	18.25	10.93	59.89%
18	18.25	6.80	37.26%

## Data Availability

Not applicable.

## Supplementary Materials

The following are available online at https://www.mdpi.com/article/10.3390/ijerph18105115/s1, Figure S1. Transport experimental device; Table S1. Measurement data and SD of zeta potential at different pH; Table S2. Measurement data and SD of zeta potential at different ionic strengths; Table S3. The meanings of model parameters; Table S4. Single-collector contact efficiency $\eta_{0}$, attachment efficiency *α* and maximum transport distance $L_{max}$ in different conditions; Table S5. Parameters for attachment–detachment model.
