# Supplementary material for: Mechanism of Stability and Transport of Chitosan-Stabilized Nano Zero-Valent Iron in Saturated Porous Media
Source: Int J Environ Res Public Health. 2021 May 12;18(10):5115. doi: 10.3390/ijerph18105115 (PMC8150866; doi:10.3390/ijerph18105115)
Supplement: Supplementary file 1 [file ijerph-18-05115-s001.zip › ijerph-1165833-supplementary.pdf]

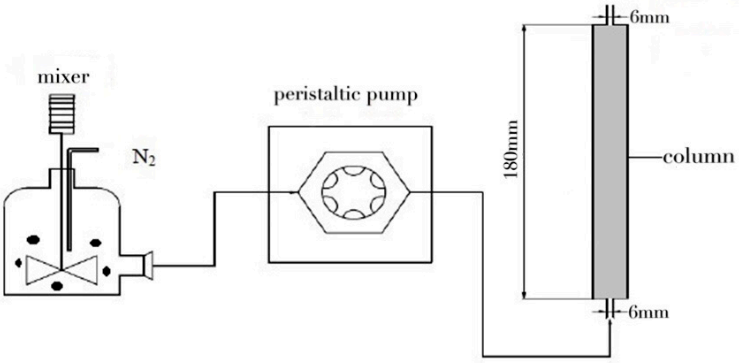

Figure S1. Transport experimental device

Table S1. Measurement data and SD of zeta potential at different pH.

	Test 1 (eV)	Test 2 (eV)	Test 3 (eV)	Mean value (eV)	SD	Lable
1	3.12	2.48	2.32	2.64	0.42332	a[1]
2	−14.42	−12.86	−13.34	−13.54	0.799	b[1]
3	−16.81	−15.52	−15.64	−15.99	0.71267	c[1]

[1] The letters a, b and c show the statistically significant differences between variables.

Table S2. Measurement data and SD of zeta potential at different ionic strength.

	Test 1 (eV)	Test 2 (eV)	Test 3 (eV)	Mean value (eV)	SD	Lable
NaCl						
1	−13	−14.3	−13.4	−13.54	0.6421	a[1]
2	−8.26	−9.32	−9.36	−8.98	0.6239	a[1]
3	−0.98	−1.29	−1.27	−1.18	0.1735	b[1]
$CaCl_2$						
1	−12.4	−13.9	−14.3	−13.54	1.00732	c[1]
2	−1.26	−0.83	−1.06	−1.05	0.21517	d[1]
3	−0.91	−0.59	−0.72	−0.74	0.16093	e[1]

[1] The letters a, b, c, d and e show the statistically significant differences between variables.

Table S3. The meanings of model parameters

Parameter	Calculation formula	Physical meaning
N_R	$\frac{d_p}{d_c}$	The ratio of particle size to that of porous media
N_{Pe}	$\frac{Ud_c}{D_\infty}$	Represents the ratio of convection to diffusion
N_{vdW}	$\frac{A}{kT}$	Van der Waals number, which represents the ratio of van der Waals effect to particle heat energy
N_{gr}	$\frac{4\pi a_p^4(\rho_p - \rho_f)g}{3kT}$	Gravity number. The ratio of gravitational potential energy to thermal energy of particles at a radius of porous media
N_A	$\frac{A}{12\pi\mu U a_p^2}$	Attraction number. It represents the comprehensive influence of van der Waals gravity and fluid velocity on the deposition rate of intercepted

particles.

N_G	$\frac{2a_p^2(\rho_p-\rho_f)g}{9\mu U}$	Gravity number, the ratio of settling velocity of Stokes particles to fluid velocity
A_S	$\frac{2(1-\lambda^5)}{(2-3\lambda+3\lambda^5-2\lambda^6)}$	Porosity related parameters of semipell model

The meanings of the parameters in the table are as follows: η_I is the contact efficiency produced by the interception mechanism; η_G is the contact efficiency caused by gravity sedimentation; η_D is the contact efficiency caused by Brownian diffusion; d_p is the particle diameter（665 nm）, d_c is the particle diameter of porous media (1.5 mm),U is the fluid approach velocity (0.0212 cm/s), D_∞ is the bulk diffusion coefficient (described by Stokes Einstein equation), A is the Hamaker constant (1.0×10^{-20} J), k is the Boltzmann constant (1.38×10^{-23} J•K^{-1}), T is fluid absolute temperature (298 K), a_p is particle radius, ρ_p is the particle density (7.86 g/cm3), ρ_f is the fluid density (1 g/cm^3), μ is the absolute fluid viscosity (0.8937×10^{-3}Pa•s^{-1}), and g is the gravitational acceleration (9.8 m• s^{-2}).

Table S4. Single–collector contact efficiency η_0、attachment efficiency α and Maximum transport distance L_{max} in different conditions

Influence factor	η_G	η_D	η_I	η_0	α	L_{max}/cm
pH = 6	0.00763	0.00144	0.00003	0.0091	1.79	71.87
pH = 7	0.00763	0.00144	0.00003	0.0091	1.15	111.89
pH = 8	0.00763	0.00144	0.00003	0.0091	1.09	118.04
0 mol/kg	0.00763	0.00144	0.00003	0.0091	1.15	111.89
0.03 mol/kg (NaCl)	0.00763	0.00144	0.00003	0.0091	2.28	56.43
0.1 mol/kg (NaCl)	0.00753	0.00144	0.00003	0.009	3.36	38.29
0.03 mol/kg ($CaCl_2$)	0.00763	0.00144	0.00003	0.0091	3.98	32.69
9 mL/min	0.00763	0.00144	0.00003	0.0091	1.15	111.89
18 mL/min	0.00353	0.00088	0.00003	0.0044	1.39	189.71

Table S5. Parameters for attachment–detachment modelMeasured / calculated parameters

**Injection rate (mL • min^{-1})**	**Permeability coefficient（cm •min^{-1}）**	**Dispersion coefficient（cm^2 • min^{-1}）**	**Saturated volume moisture content**	**Sand bulk density（g •cm^{-3}）**	**Particle deposition rate coefficient（min^{-1}）**
9	4.64	0.43	0.41	1.5	0.192
18	4.64	0.43	0.41	1.5	0.114

Calibration parameters

Injection rate（mL • min^{-1}）	Straining coefficient（min^{-1}）	Detachment coefficient（min^{-1}）	Fitting parameters of spatial distribution
9	1.88	0.01	0.43
18	3.8	0.06	0.43
